# A Rare Case of Pancreatic Schwannoma

**DOI:** 10.7759/cureus.25688

**Published:** 2022-06-06

**Authors:** Anastasia Tambovtseva, Bilal Ashraf, Saud E Suleiman, Ziad Suleiman, Ziad Alaidy

**Affiliations:** 1 Internal Medicine, University of Central Florida College of Medicine, Ocala, USA; 2 Internal Medicine, HCA North Florida, Ocala, USA; 3 Advanced Gastroenterology, Halifax Health Medical Center, Daytona Beach, USA; 4 Gastroenterology, Florida State University College of Medicine, Daytona Beach, USA; 5 Biology, University of Florida, Gainesville, USA; 6 Breast Cancer Research, John’s Hopkins Hospital, Baltimore, USA

**Keywords:** pancreatic mass, rare tumor, pancreatic tumor, pancreatic schwannoma, schwannoma

## Abstract

Pancreatic schwannoma is a neuroendocrine cell tumor that arises in the pancreas. It is very rare, and, to date, only fewer than 70 similar cases have been reported in the literature. Here, we present another case of this type of tumor in a 68-year-old female.

In addition to describing the pancreatic schwannoma, we discuss the major challenges associated with its diagnosis and management. As such, clinically and on imaging, pancreatic schwannomas are almost indistinguishable from other cancerous or benign pancreatic tumors. Therefore, only a biopsy can definitively diagnose pancreatic schwannomas by demonstrating spindle-shaped cells with immunohistochemistry positive for S-100.

Because pancreatic schwannomas are very rare, it is important to increase awareness among clinicians about this condition and inform them regarding the challenges associated with its diagnosis and management.

## Introduction

Different types of malignant and benign tumors are seen in the pancreas. Schwannoma is among the very rare tumors that may arise in the pancreas. Schwannomas most commonly occur in neuron-rich areas such as the brain or back because these tumors consist of Schwann cells that make a myelin sheath for neuronal axons. However, even though rare, schwannomas can also be seen in the pancreas. The prevalence of pancreatic schwannomas appears to be extremely low as currently in the literature only fewer than 70 cases have been reported [1]. Even though this type of tumor is very rare, it is important to consider it when going through differential diagnoses for pancreatic mass. Because pancreatic schwannomas may present clinically similar to other pancreatic cancers and tumors, and it is difficult to distinguish it on images from other tumor types, biopsy or surgery with further pathology analysis can confirm the diagnosis. As the majority of pancreatic cancers are among the most aggressive cancers and require immediate treatment, and a small percentage of pancreatic schwannomas may also be malignant, timely diagnosis with biopsy and possible surgery should not be delayed. Increasing awareness of clinicians about this rare condition can guide diagnostic and management plans.

## Case presentation

**Figure 1:**
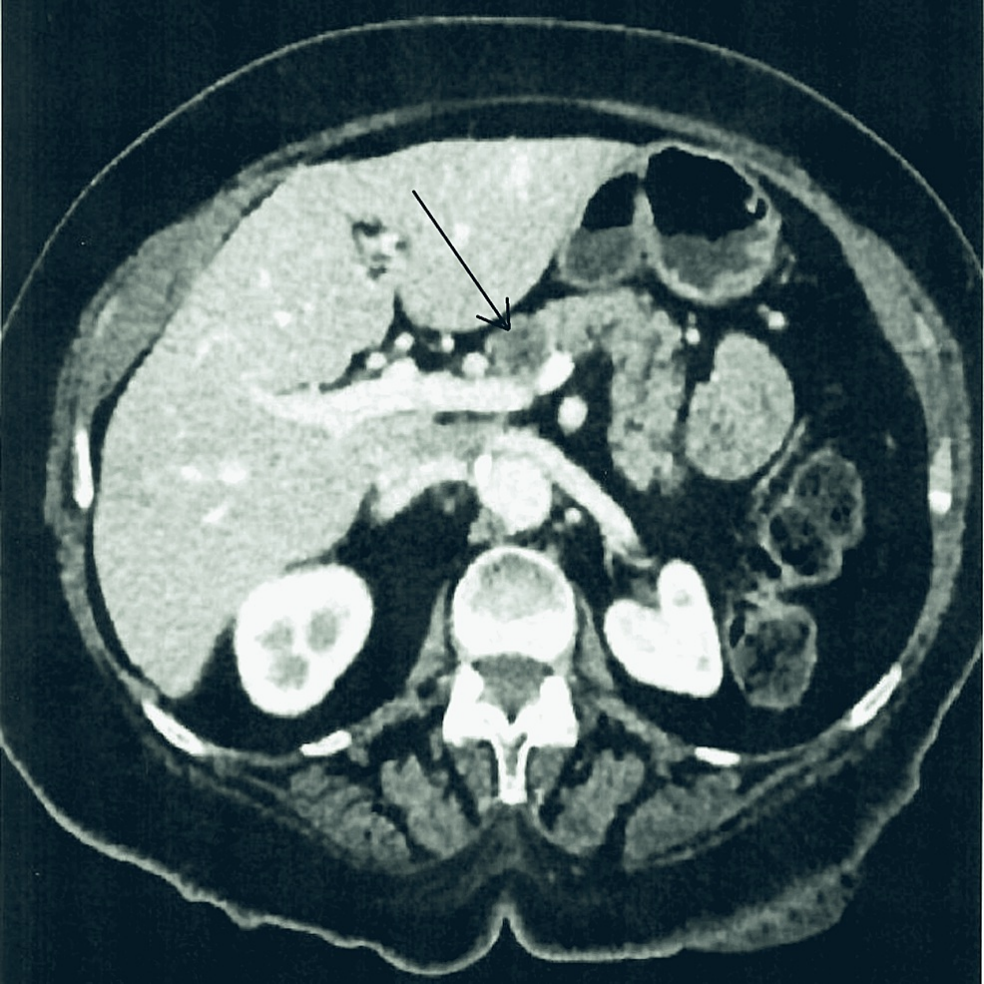
Pancreatic schwannoma on computed tomography scan of the abdomen (arrow).

**Figure 2:**
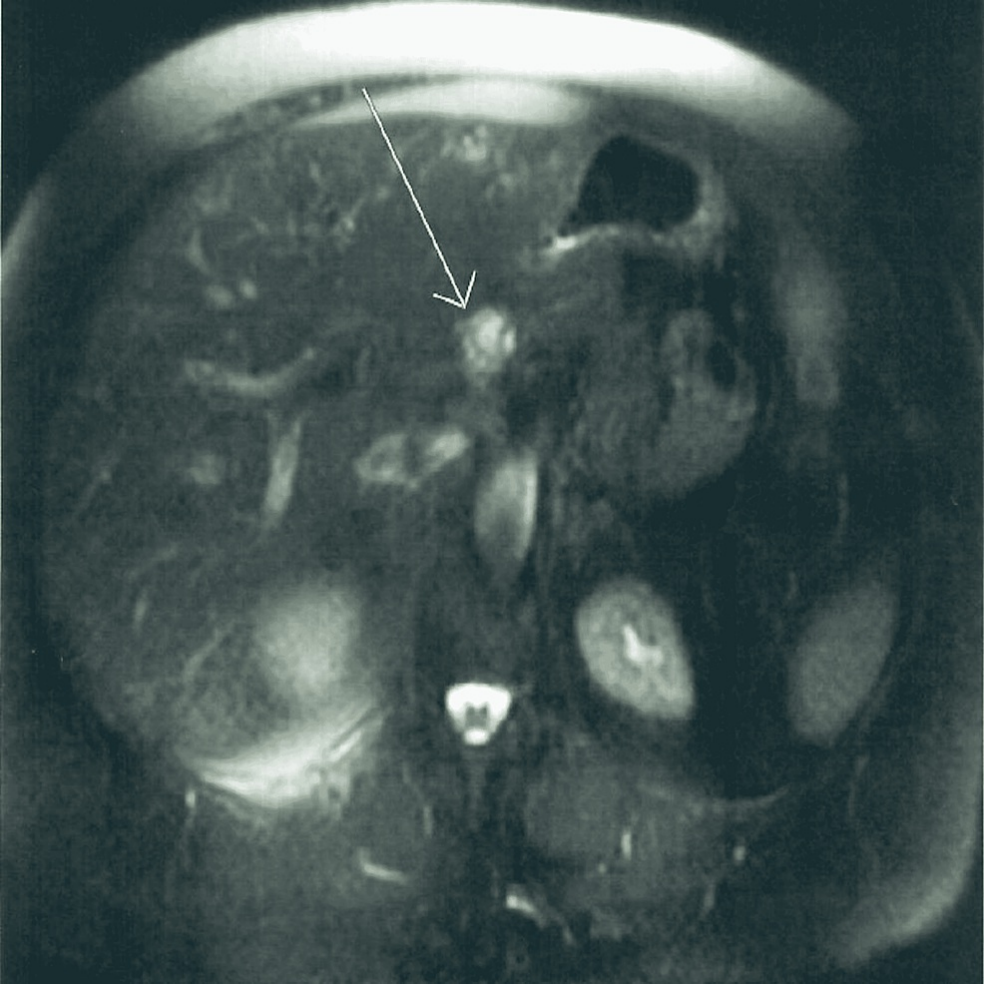
Pancreatic schwannoma on magnetic resonance imaging of the abdomen (arrow).

**Figure 3:**
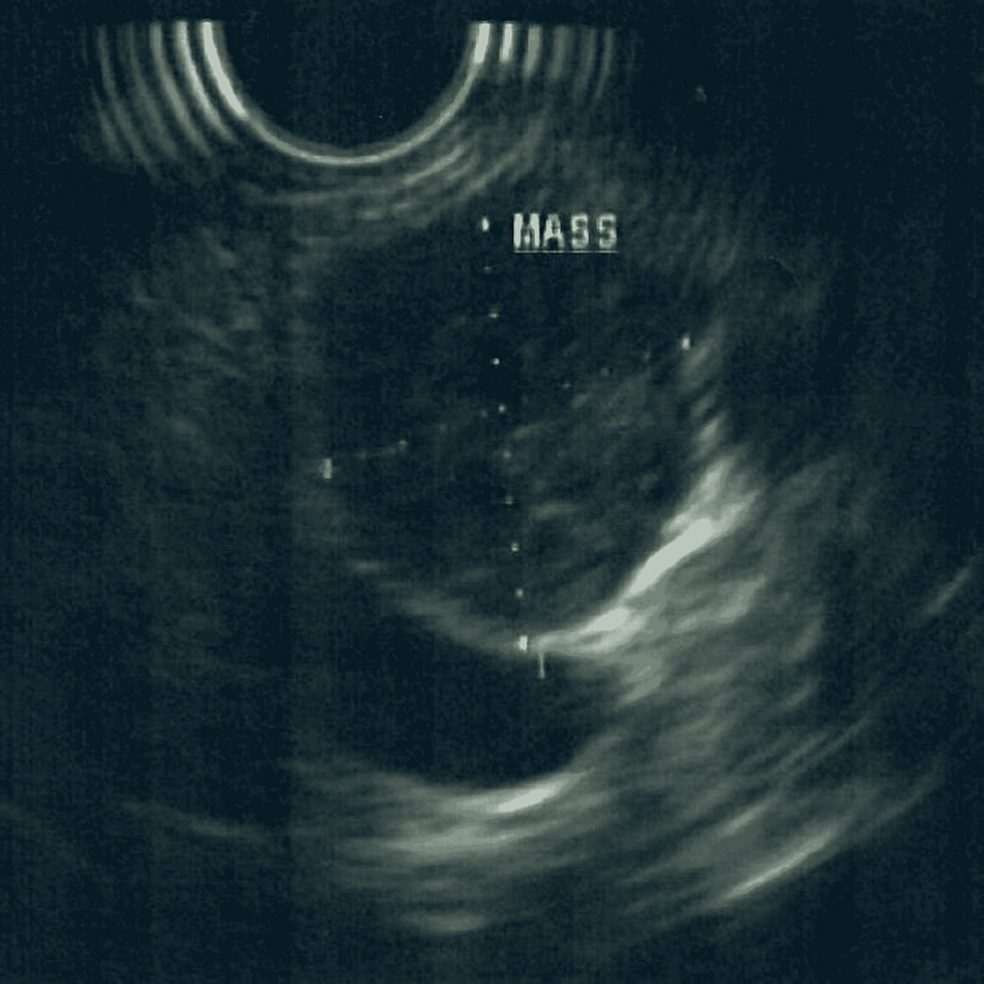
Partially cystic and solid pancreatic schwannoma on endoscopic ultrasound without Doppler.

**Figure 4:**
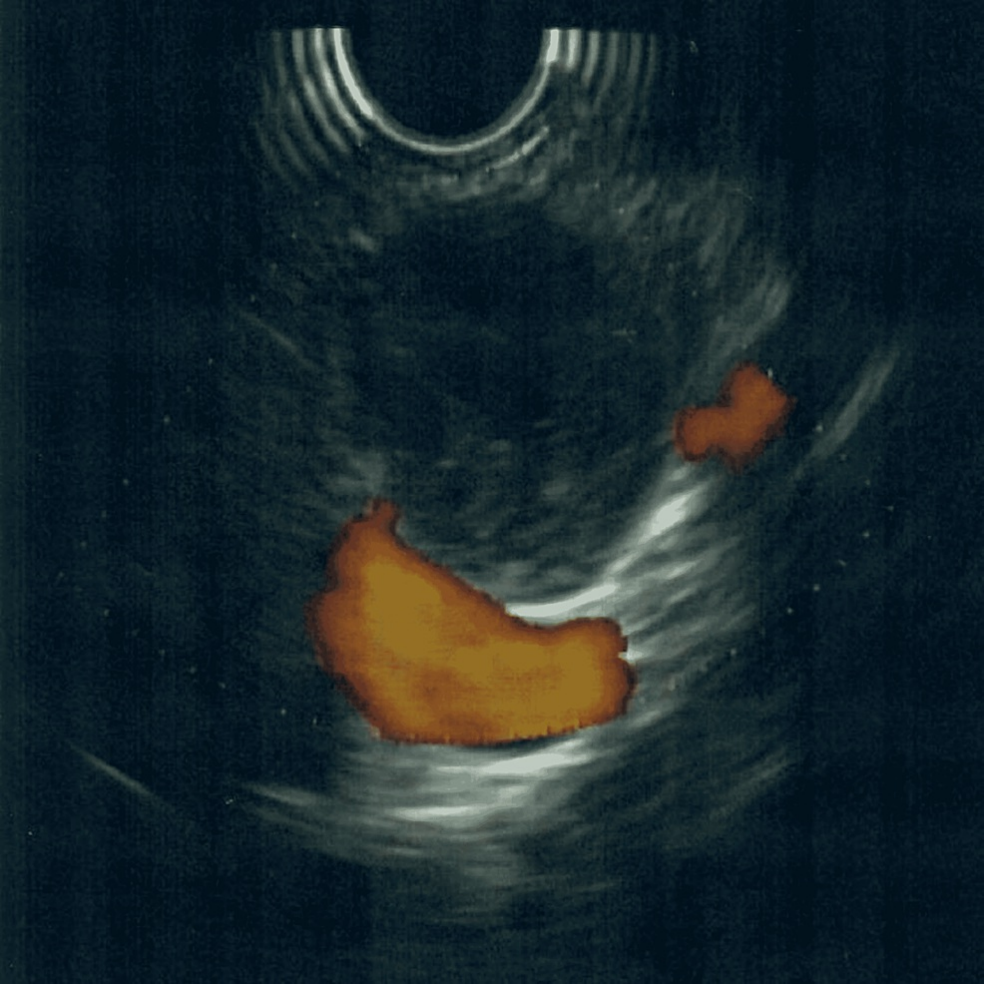
Partially cystic and solid pancreatic schwannoma on endoscopic ultrasound with Doppler.

A 68-year-old female presented with a complaint of chest and epigastric pain. After a negative cardiac workup, a computed tomography (CT) scan of the abdomen was done and showed a 19 mm pancreatic neck mass with subtle contrast enhancement concerning for a neoplasm (Figure 1). Magnetic resonance imaging (MRI) revealed a 17 mm partially cystic and solid mass at the head and neck of the pancreas, with no identified lymphadenopathy or ductal dilatation (Figure 2). The mass was further investigated with endoscopic ultrasound, which confirmed the mass. A fine-needle aspiration biopsy was performed (Figures 3, 4).

**Figure 5:**
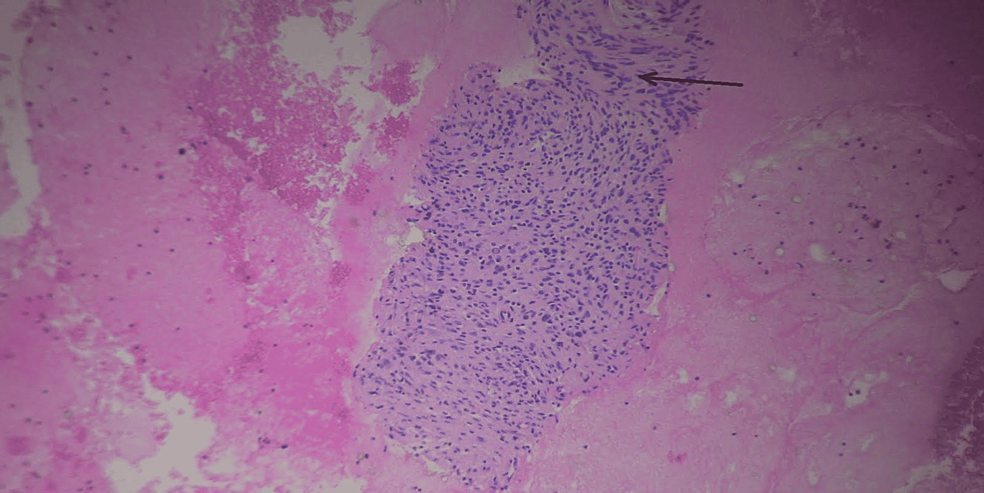
Hematoxylin and eosin stain showing fragments of bland-appearing spindle cells without mitotic activity or necrosis.

**Figure 6:**
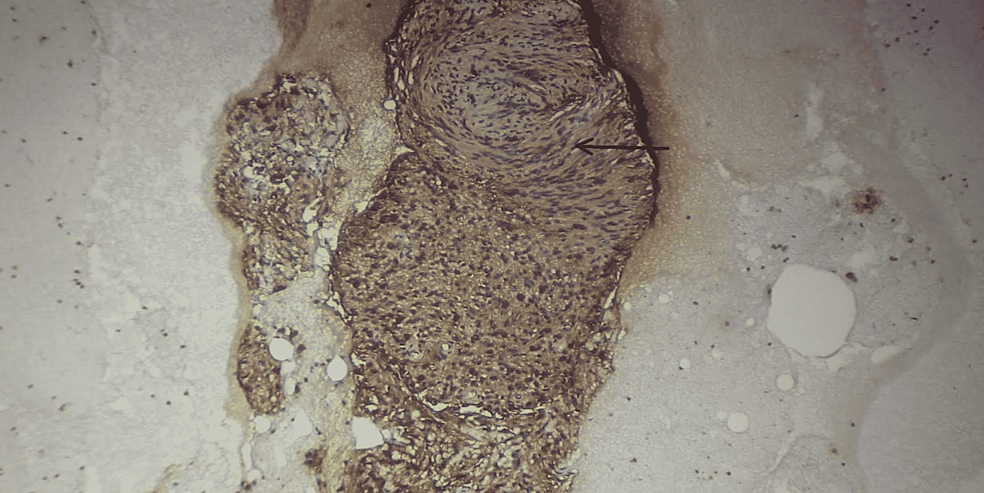
Immunohistochemistry showing S-100-positive tumor cells.

The pathological report revealed a tumor composed of multiple fragments of bland-appearing spindle cells without mitotic activity or necrosis (Figure 5). Immunohistochemical stain showed tumor cells to be positive for S-100, and a diagnosis of pancreatic schwannoma was made (Figure 6).

## Discussion

Pancreatic schwannoma is a very rare pancreatic tumor that accounts for 1% of other schwannomas [2]. The majority of pancreatic schwannomas are benign, but a small number of them may be malignant [1]. There are challenges associated with diagnosing this type of tumor. Clinically, pancreatic schwannoma is indistinguishable from other types of pancreatic tumors. Patients may either be completely asymptomatic or may present with abdominal, chest, or back pain [1-3]. This is similar to other pancreatic tumors. Imaging modalities, including CT, MRI, and endoscopic ultrasound, can help to identify the pancreatic mass but cannot distinguish pancreatic schwannoma from other tumor types of the pancreas because of its wide range of morphologies [4]. Pancreatic schwannoma might appear as a solid, cystic, or as compound lesion [2,5-7]. The majority of pancreatic schwannomas appear to be in the head and neck of the pancreas, but a small percentage may also be located in the tail of the pancreas [8]. Schwannoma may be mistaken for various benign and malignant pancreatic tumors [3]. When pancreatic schwannoma appears as a mixed lesion of solid and cystic components, it resembles malignant pancreatic tumors and can be perceived that way even though pancreatic schwannoma is benign in the majority of cases. Some studies have shown that pancreatic schwannomas are also highly metabolic and light up on positron emission tomography-computed tomography scans, which also might be mistaken for malignancy [4,9]. This similarity might pose a risk for possible misdiagnosis and inappropriate management in some cases. Therefore, a biopsy is essential to establish the correct diagnosis. Management of pancreatic schwannomas may also be somewhat challenging. It can successfully be managed with surgery in severe symptomatic cases [10], and in the majority of cases, definitive treatment is needed. However, even though the majority of pancreatic schwannomas are benign, there is a very minimal risk of malignancy [7,11]. Biopsy in this case may help to confirm a diagnosis. Moreover, according to patient risk versus benefit, surgery may be avoided in some cases [12,13]. Increasing awareness of this condition is crucial. Knowing that pancreatic schwannoma can resemble malignant pancreatic tumors can help clinicians to make decisions regarding the management of patients with pancreatic masses and avoid misdiagnoses.

## Conclusions

A pancreatic schwannoma is a very rare tumor. This case report focuses on increasing awareness of this type of pancreatic tumor among healthcare providers and discusses the challenges associated with its diagnosis.
